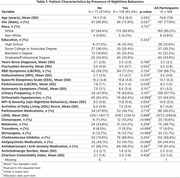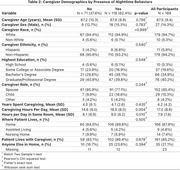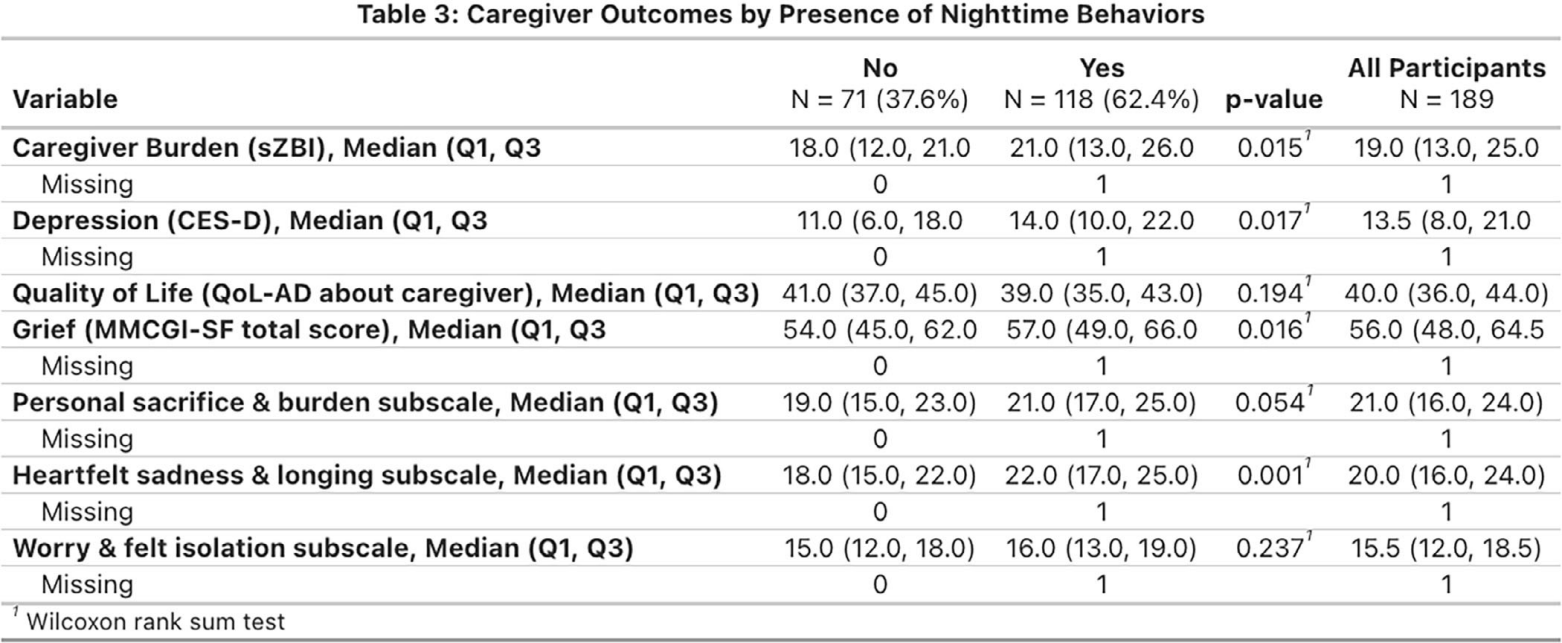# Sleepless nights: Caregiver burden among DLB patients with nighttime behaviors

**DOI:** 10.1002/alz70861_108669

**Published:** 2025-12-23

**Authors:** Jesse S Cohen, Zhigang Li, Tingchang Wang, Melissa J. Armstrong

**Affiliations:** ^1^ University of Florida, Jacksonville, FL USA; ^2^ University of Florida, Gainesville, FL USA; ^3^ Fixel Institute for Neurological Diseases, Gainesville, FL USA

## Abstract

**Background:**

Comorbid neuropsychiatric and sleep disorders are common in dementia with Lewy bodies (DLB), often resulting in nocturnal agitation. However, the impact of nighttime behaviors on caregiver burden remains poorly described.

**Method:**

Baseline data were used from PACE‐DLB (Predicting ACcurately End‐of‐Life in Dementia with Lewy Bodies and Promoting Quality End‐of‐Life Experiences), a longitudinal study of moderate to advanced DLB patient‐caregiver dyads. Individuals were divided into patients with and without nocturnal behaviors based on Neuropsychiatric Inventory Questionnaire (NPI‐Q) question 11 regarding nighttime behaviors with severity = moderate/severe. The primary outcome was severity of caregiver burden as measured by the shortened Zarit Revised Burden interview (sZBI), using the Wilcoxon rank sum test to compare groups.

**Result:**

Of 189 participants, 118 (62.4%) had nighttime behaviors (NTB+), while 71 (37.6%) did not (NTB‐). The groups were generally well matched for age (mean 75.4±8.0 in NTB+, 74.1±7.4 in NTB‐, *p* =0.26), sex (86 males (72.9%) in NTB+, 61 males (85.9%) in NTB‐, *p* =0.06) and education (*p* =0.24). The NTB+ group scored higher (worse) in fluctuation severity (CAF 3.4±0.7 vs. 3.0±0.9, *p* =0.002), daytime sleepiness (ESS 15.8±4.5 vs. 10.2±4.8, *p* ≤0.001), parkinsonism (LBCRS 8.3±1.4 vs. 7.8±1.7, *p* =0.038), neuropsychiatric symptoms (NPI‐Q 11.8±5.8 vs. 9.2±4.6, *p* =0.004), and activities of daily living (7.2±4.4 vs. 6.0±4.8, *p* =0.035). There were no differences in disease duration, dementia severity, autonomic symptoms (including urinary frequency), RBD, or medications. There were no significant differences in caregiver demographics. Caregivers of NTB+ spent more hours caregiving per day (18.5±8.0 vs 14.6±9.0, *p* =0.004) and more hours in the same room as the patient (9.0±8.2 vs 6.1±6.9, *p* =0.01). Caregiver burden was greater in NTB+ (sZBI 21 [IQR13‐26] vs 18 [IQR12‐21], *p* =0.015), with higher levels of depression and grief among caregivers of NTB+ patients.

**Conclusion:**

Nighttime behaviors in individuals with DLB associates with increased caregiver burden. The presence of these behaviors should prompt providers to screen for caregiver burden and depression. Further study is needed to determine the extent to which this effect is mediated by disrupted caregiver sleep.